# The neurochemical substrates of habitual and goal-directed control

**DOI:** 10.1038/s41398-020-0762-5

**Published:** 2020-03-03

**Authors:** Valerie Voon, Juho Joutsa, Joonas Majuri, Kwangyeol Baek, Camilla L. Nord, Eveliina Arponen, Sarita Forsback, Valtteri Kaasinen

**Affiliations:** 1 Department of Psychiatry, University of Cambridge, Cambridge, UK; 2 Cambridgeshire and Peterborough Foundation NHS Trust, Cambridge, UK; 3 NIHR Biomedical Research Centre, Cambridge University, Cambridge, UK; 4 Clinical Neurosciences, University of Turku, Turku, Finland; 5 Turku Brain and Mind Center, University of Turku, Turku, Finland; 6 Turku PET Centre, Turku University Hospital, Turku, Finland; 7 Division of Clinical Neurosciences, Turku University Hospital, Turku, Finland; 8 School of Biomedical Convergence Engineering, Pusan National University, Busan, Republic of Korea; 9 MRC Cognition and Brain Sciences Unit, University of Cambridge, Cambridge, UK

**Keywords:** Neuroscience, Human behaviour

## Abstract

Our daily decisions are governed by the arbitration between goal-directed and habitual strategies. However, the neurochemical basis of this arbitration is unclear. We assessed the contribution of dopaminergic, serotonergic, and opioidergic systems to this balance across reward and loss domains. Thirty-nine participants (17 healthy controls, 15 patients with pathological gambling, and 7 with binge eating disorder) underwent positron emission tomography (PET) imaging with [^18^F]FDOPA, [^11^C]MADAM and [^11^C]carfentanil to assess presynaptic dopamine, and serotonin transporter and mu-opioid receptor binding potential. Separately, participants completed a modified two-step task, which quantifies the degree to which decision-making is influenced by goal-directed or habitual strategies. All participants completed a version with reward outcomes; healthy controls additionally completed a version with loss outcomes. In the context of rewarding outcomes, we found that greater serotonin transporter binding potential in prefrontal regions was associated with habitual control, while greater serotonin transporter binding potential in the putamen was marginally associated with goal-directed control; however, the findings were no longer significant when controlling for the opposing valence (loss). In blocks with loss outcomes, we found that the opioidergic system, specifically greater [^11^C]carfentanil binding potential, was positively associated with goal-directed control and negatively associated with habit-directed control. Our findings illuminate the complex neurochemical basis of goal-directed and habitual behavior, implicating differential roles for prefrontal and subcortical serotonin in decision-making across healthy and pathological populations.

## Introduction

Two distinct systems influence our choice behavior: goal directed and habitual control. Goal-directed (or model-based) control is characterized by a learned internal model of the environment that can dynamically evaluate optimal actions, a flexible but computationally expensive strategy^1–3^. By contrast, habitual (or model-free) control computes the value of each action entirely by past experience (reward prediction errors), sacrificing flexibility for greater efficiency. Disruptions in the balance of these strategies may underlie a range of pathological behaviours, in particular psychiatric disorders characterized by compulsivity^3–5^.

This balance between goal-directed and habitual strategies is mediated by various neurochemical processes. Among these, the dopamine system is most frequently implicated; a smaller number of studies also point to the involvement of the serotonin and opioid systems^3,6^. The role of dopamine in this balance is a topic of some debate. Traditionally, dopamine has been associated with model-free reinforcement learning: in rodents, pharmacologically enhancing dopamine increases habit formation^7^, while dopaminergic nigrostriatal lesions impair habit formation^8^. However, more recent human research has shown that depleting dopamine *increases* habitual control^9^, while administration of the dopamine precursor levodopa was reported to enhance goal-directed control in two studies^10,11^ and reduce habitual control in a third^12^ (in the latter study, participants with high working memory capacity did show enhancement of goal-directed control). There is evidence that a key locus of this influence is the ventral striatum: a study that combined 6-[^18^F]fluoro-L-dopa ([^18^F]FDOPA) positron emission tomography (PET) with functional magnetic resonance imaging found goal-directed learning correlated with ventral striatal presynaptic dopamine synthesis capacity^13^. In line with this work, we expected that heightened dopamine levels might shift decision-making toward a goal-directed and away from a habitual strategy. However, most previous work has focused exclusively on choice behavior in the reward domain^14–16^, a crucial limitation, making the involvement of dopamine in the loss domain unclear. Thus, probing the neurochemical substrates of model-based and model-free control across reward and loss domains may yield a fuller picture of the neural basis of decision-making.

The opioid and serotonin systems appear to play a role in arbitrating between goal-directed and habitual control of behaviour. In rodents, decreasing forebrain serotonin (5-HT) increases compulsive cocaine seeking and manipulating the serotonergic system shifts these habitual behaviours^16^. Overexpression of rodent dorsolateral striatal 5-HT6 receptors also decreases habitual control^15^. In healthy humans, central serotonin depletion enhances habitual responding^17^. However, central serotonin depletion impairs goal-directed control to rewards, but enhances goal-directed control to losses^6^, illustrating the importance of including both reward and loss domains experimentally. The opioid system also plays an essential role in goal-directed behaviour. A large body of evidence implicates the opioid system in goal-directed aspects of reward processing: opioid peptide-containing neurons, their terminals, and opioid receptors are present in the same basal forebrain regions implicated in learning and performance of goal-directed actions (e.g., the nucleus accumbens (NAcc) core)^18,19^.

Compellingly, in rodents, blockade of the opioid system during learning with naloxone compromises goal-directed learning, enhancing habitual control of actions^14^. Naloxone administration also decreases goal-directed alcohol consumption in an animal model of alcoholism, and blocks reinstatement of alcohol-seeking learned in a goal-directed schedule^20^. Opioid processes seem critical for the acquisition of normal goal-directed control of actions: potentially, higher endogenous opioid levels would have the opposite effect to naloxone administration, enhancing goal-directed control of actions.

Here, we investigate the balance of goal-directed (model-based) and habitual (model-free) control in the appetitive and aversive domain (monetary rewards and losses), and its relationship with NAcc and ventromedial prefrontal cortex (vmPFC)/medial orbitofrontal cortex (mOFC) presynaptic dopamine function, and serotonin transporter (SERT) and mu-opioid receptor (MOR)-binding potential (BP). Previous studies investigating dopamine or serotonin function in association with model-free/model-based control have primarily focused on the striatum (e.g.,^13,15^). We additionally include a vmPFC/mOFC ROI, due to previous work suggesting the vmPFC is involved at least in part in model-based evaluation in this task^2^. Moreover, in healthy populations, lower medial OFC and vmPFC volumes (as well as striatal volumes) are associated with reduced model-based control^4^, while reduced medial prefrontal cortex activation during model-based control is predictive of relapse in alcohol-dependent patients^21^, underlining the clinical relevance of this region’s computations during the task.

We include three populations of subjects: healthy controls, patients with pathological gambling (PG), and those with binge-eating disorder (BED); in both BED and addictive disorders, decision-making is shifted away from goal-directed toward habitual control (and is thought to be a transdiagnostic symptom dimension common across disorders of compulsivity)^4^. However, the primary purpose of this study was not to assess between-group differences, which we explored separately^22^, but rather to illuminate the role of these three neurochemical systems (dopamine, serotonin, and opioid) in goal-directed and habitual control, across reward and loss domains. Thus, we included psychiatric populations in our sample in order to capture a wider range of goal-directed and habitual behavior (associated with healthier and pathological states, respecitvely). We hoped this approach would yield greater insight into the neurochemical substrates of this behaviour.

We hypothesized that heightened [^18^F]FDOPA uptake (signifying greater pre-synaptic dopamine function) would be associated with heightened goal-directed learning to rewards; that lower [^11^C]MADAM BP (which binds selectively to the SERT) would be associated with decreased goal-directed control; and that lower [^11^C]carfentanil BP (which binds to the MOR) would be associated with decreased goal-directed control.

## Materials and methods

### Participants

Sixty-seven prospective participants were screened for the study. Subjects recruited to BED and PG groups fulfilled the Diagnostic and Statistical Manual of Mental Disorders (DSM-IV) criteria for BED and PG, respectively, confirmed in a structured clinical interview. Exclusion criteria common to both groups, as well as healthy volunteers, included any substance use disorder during the last 6 months prior to PET imaging, diagnosed DSM-IV axis I psychiatric disorder, any clinically relevant somatic disorder (e.g., diabetes mellitus), pregnancy or lactation, and weight over 180 kg (the scanner limit). After screening, 17 healthy controls, 15 PG patients, and 7 BED patients were recruited to the study. The study protocol was approved by the local ethical committee, and all participants gave written informed consent. We required 36 subjects to detect a large effect size (*f*^2^ = 0.3) with 80% power (G*Power: Linear multiple regression). The study was conducted according to the principles of the Declaration of Helsinki.

### Two-step task

**Fig. 1:**
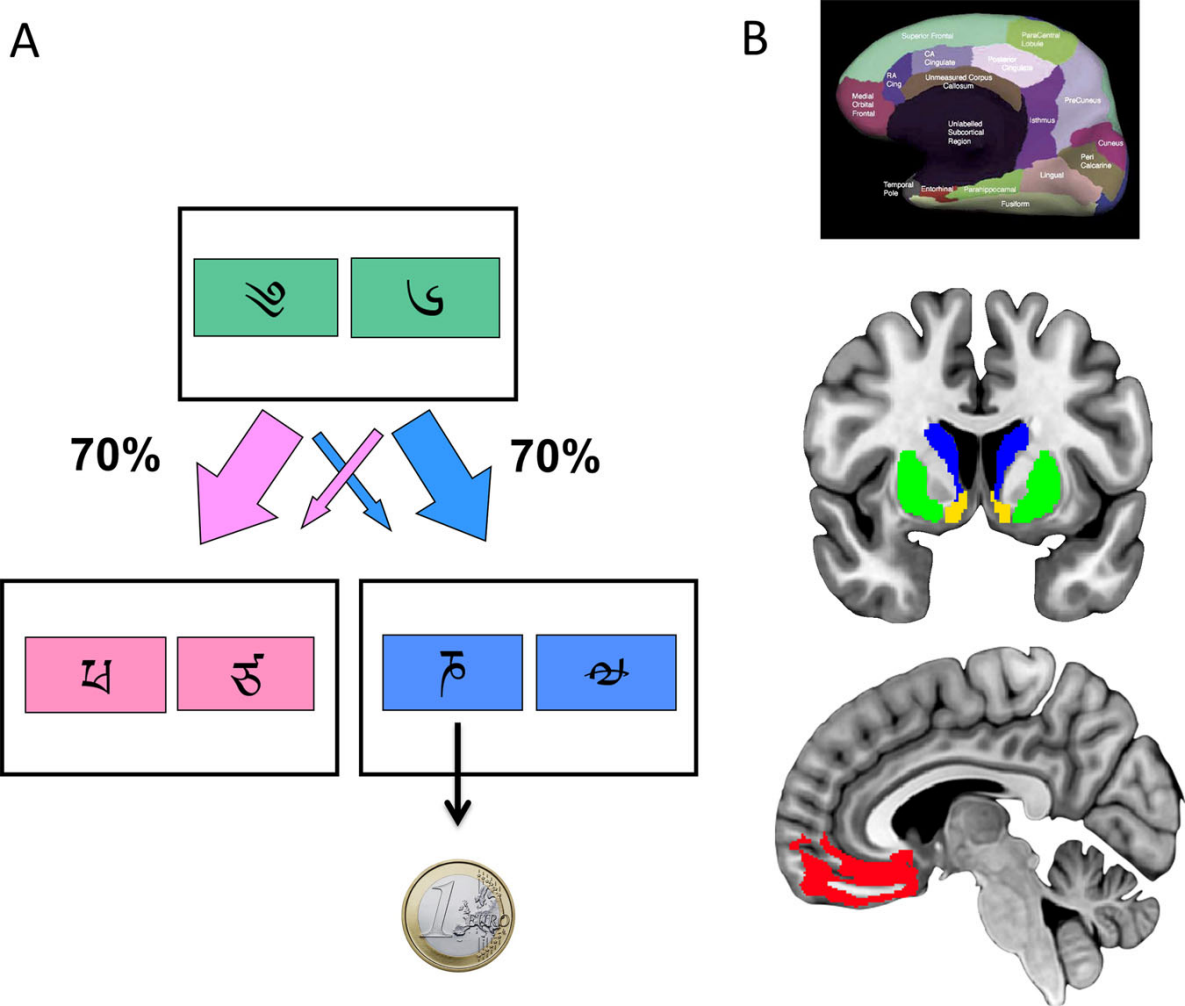
Two-step task and regions of interest. **a** Subjects choose between a stimuli-pair at the first stage which leads with fixed probability (*p* = 0.70) to one of two states at the second stage. Subjects then choose between one of two stimuli-pairs at the second stage which are associated with a shifting probability of reward based on a random Gaussian walk. The reward outcome is shown. **b** Region of interests Top: freesurfer parcellation. Middle and bottom: Regions of interest. The caudate is shown in blue; the putamen is shown in green; the nucleus accumbens (ventral striatum) is shown in yellow. The medial orbitofrontal cortex parcellation shown in the Free Surfer parcellation (top) consisted of both the ventromedial prefrontal cortex and medial orbitofrontal cortex (shown in red at the bottom). For illustration purposes, the Free Surfer reconstruction is shown overlaid on the MNI152 brain (middle and bottom).

Healthy participants performed the two-step task in two conditions, monetary reward or loss; all patient groups performed only the reward version of the task. We have previously described the task^4,23^. Briefly, the task consisted of two stages (see Fig. 1a). In stage 1, participants chose between two stimuli, each of which led to one of two stimulus pairs with a fixed probability (*p* = 0.70) and to the other stimulus pair with opposite probability (*p* = 0.30). In stage 2, participants chose a single stimulus from the resulting pair; this choice led to an outcome.

Each of the four stimuli in stage 2 was attached to a different probability distribution, with probability varying slowly and independently over time between 0.25 and 0.75. The association between each stage 2 stimulus and its reward probability was counterbalanced across participants. Choices at each stage had to be made within 2 s, and the result of each choice was presented for 1 s, after a 1.5 s delay. The stimuli chosen in stages 1 and 2 remained on screen as a reminder in stage 2 and the outcome stage, respectively. If the stage 2 choice was rewarded, participants saw a 1 Euro coin for 1 s; otherwise, they saw a grey circle for 1 s. In the reward condition, subjects either saw a 1 Euro coin with a green square (win outcome), or a grey circle (no-win outcome). In the loss condition, subjects either saw a 1 Euro coin with a red square and red cross over the coin (loss outcome), or a grey circle (no-loss outcome).

The task consisted of two blocks of 67 trials each per condition. The order of the conditions was randomized (but the two blocks of each condition were always run sequentially). Prior to the task, participants underwent extensive computer-based instructions, which included explanatory examples of changes in transition and probability, and a short block of 50 trials in the same format as the experimental task but with different stimuli. The task was run with Cogent 2000 (http://www.vislab.ucl.ac.uk/cogent.php) on Matlab R2011a (Mathworks, Natick, USA). See Supplemental Materials for an analysis on existing datasets comparing this shortened (two-block) version of the task with the typical three-block version: we showed that the average main outcome measure was highly correlated between the two versions.

### PET imaging

All subjects underwent PET scanning three times: first using the MOR-ligand [^11^C]carfentanil, then with the SERT-ligand [^11^C]MADAM, and finally with the dopamine precursor ligand [^18^F]FDOPA. The syntheses of these tracers have been described in detail previously^22,24^. The PET imaging was performed with an high resolution research tomograph (Siemens Medical Solutions, Knoxville, TN, USA) PET scanner used in 3D list mode with scatter correction. A transmission scan was performed before each PET scan with a [^137^Cs] rotating point source. The dynamic scanning times were 51, 90, and 90 min for [^11^C]carfentanil, [^11^C]MADAM, and [^18^F]FDOPA, respectively. All three PET scans were conducted in the same day at fixed intervals: [^11^C]carfentanil scan at 0900–1000 h, regular hospital lunch at 1100–1200 h, [^11^C]MADAM scan at 1200–1300 h and [^18^F]FDOPA scan at 1430–1530 h. One [^11^C]carfentanil scan and three [^18^F]FDOPA scans were performed on a separate day due to tracer production failure or scanner malfunction. Head movements were minimized using a personalized thermoplastic mask or a Velcro strap, and recorded with a stereotaxic infrared camera (Polaris Vicra, Northern Digital, Waterloo, Canada). One [^11^C]carfentanil scan, three [^18^F]FDOPA scans and three [^11^C]MADAM scans were excluded due to scanner malfunction or subject withdrawal. Thus, the final sample sizes were 7 BED, 15 PG, and 16 controls with [^11^C]carfentanil, 7 BED, 13 PG, and 16 controls with [^11^C]MADAM and [^18^F]FDOPA.

The preprocessing and analysis has been described in detail previously^22^. Briefly, PET images were corrected for between-frame motion and coregistered with individual anatomical T1-weighted magnetic resonance imaging (MRI) using Statistical Parametric Mapping software (SPM8, http://www.fil.ion.ucl.ac.uk/spm/software/spm8/). Time-activity data were extracted using regions of interest (ROI) for the mean NAcc area, caudate, putamen and mOFC, which were determined from the individual T1-weighted MR images using FreeSurfer automatic parcellation (Fig. 1b, top) (version 5.3.0, http://surfer.nmr.mgh. Harvard.edu/) as described earlier^25–27^. Note that the automated mOFC ROI includes both vmPFC and mOFC regions and is referred to in this study as vmPFC/mOFC (Fig. 1b). The simplified reference tissue model was applied to calculate [^11^C]carfentanil and [^11^C]MADAM estimates of specific binding relative to non-displaceable BPs (BP_ND_)^28^. [^18^F]FDOPA influx rate constant (*K*_i_) was determined using the Patlak plot using the reference region as the input function^29^. The occipital cortex was designated as the reference region for [^11^C]carfentanil and [^18^F]FDOPA, and the cerebellar cortex was the reference region for [^11^C]MADAM. Different reference regions ensure there is no specific tracer binding in the reference region (in the case of [^11^C]MADAM, there is specific binding in the occipital cortex but no specific binding in the cerebellar cortex^30^; for [^11^C]carfentanil and [^18^F]FDOPA, there is no specific binding in the occipital cortex^31,32^).

### Analysis

All PET data were tested for outliers (>3 standard deviation (SD) from group mean) and normality of distribution (Shapiro Wilkes test *p* > 0.05). The computational analysis for the two-step task has been extensively described previously^4,33^. In brief, we fit choice data of each participant to a hybrid algorithm that combined model-free (i.e., reinforcement learning) and model-based learning algorithms. This model estimates five parameters based on the behavioural data for each participant: a choice reliability parameter (*β*) a learning rate (*α*), a reinforcement eligibility parameter (*λ*), a perseveration rate, and a weighting parameter (*w*, which extends from 1 (purely model-based) to 0 (purely model-free). We analyse only this final parameter, described as *w*_*r*_ = *w* for the reward condition, and *w*_*l*_ = *w* for the loss condition.

Two healthy controls did not complete the two-step task for loss outcomes. We tested *w*_*r*_ and *w*_*l*_ for outliers (>3 SD from group mean) and normality of distribution (Shapiro Wilkes test *p* > 0.05). As the scores were normally distributed we used parametric analyses. We compared *w*_*r*_ between groups in the behavioural analysis using a one-way ANOVA (but did not conduct any group comparisons for *w*_*l*_ as only healthy volunteers were tested in the loss condition). For the relationship with neural regions associated with the PET ligands, we conducted six stepwise multiple linear regressions with backwards elimination, with either *w*_*r*_ or *w*_*l*_ as the dependent variable and the mean bilateral NAcc, caudate, putamen and vmPFC/mOFC of each PET ligand data as the independent variables (no multicollinearity was detected with VIF < 10; homoscedascity of residuals and normality of residuals were confirmed). The *w*_*r*_ analysis included healthy controls, PG, and BED; since only healthy controls were tested in the loss condition, the *w*_*l*_ model included only healthy controls. For these models, *p* < 0.0083 was considered significant (after Bonferroni correction for six regression analyses: one model for each ligand, for both reward and loss).

## Results

We assessed 17 healthy controls, 15 patients with PG, and 7 patients with BED (see Table 1 for demographic details, and see previous publications for additional clinical details^22,34^). Age did not differ between groups (*p* = 0.35), though there was a group effect of body mass index (BMI) (*p* = 0.003, driven by an increased BMI in the BED population) and on the Beck Depression Inventory (BDI) (*p* < 0.0005, driven by higher BDI scores in both patient populations). There were also group differences across all gambling measures (driven by higher scores in the PG group) and binge eating measures (driven by higher scores in the BED group); all *p* < 0.01 (see Table 1).

**Table 1:** Demographic details of the participants.

Measure	Healthy controls (*N* = 17)	BED (*N* = 7)	PG (*N* = 15)
Mean age (SD)	43.29 (11.10)	49.43 (5.09)	42.60 (11.81)
Males	8	0	8
Mean BMI (SD)	24.82 (2.10)	30.87 (6.58)	25.41 (3.64)
Mean BDI (SD)	2.82 (3.09)	15.43 (9.62)	14.36 (7.76)
SOGS	0.1 (0.3)	0.4 (0.5)	13.3 (2.3)
Duration of problem gambling (y)	n.a.	n.a.	11.6 (7.3)
Gambling per week (€)	3.9 (7.4)	2.9 (4.6)	152 (149)
Gambling per week (h)	0.5 (1.2)	0.5 (1.2)	8.7 (7.2)
Gambling debt (€)	0 (0)	0 (0)	18,000 (15,600)
Binge eating scale	2.1 (2.1)	30.9 (4.6)	4.4 (4.4)
Yale food addiction scale	5.4 (3.4)	42.3 (6.5)	9.1 (9.5)
DEBQ emotional	20.5 (5.0)	50.0 (8.3)	21.2 (8.7)
DEBQ external	23.7 (5.3)	37.5 (6.3)	26.1 (7.3)
DEBQ restrained	24.8 (6.8)	35.3 (3.4)	20.9 (10.6)
Duration of problem eating (y)	n.a.	18.1 (14.9)	n.a.

*SD* standard deviation, *BED* Binge eating disorder, *PG* pathological gambling, *BMI* body mass index, *BDI* Beck depression inventory, *DEBQ* the Dutch eating behavior questionnaire, *SOGS* south oaks gambling screen, *n.a.* not applicable.

We first analysed the behavioural results alone to test if the groups differed on measures of model-based and model-free control on *w*_*r*_ (extracted from the computational model that putatively describes the degree of model-based or model-freeness of a subject). There were no significant group differences in *w*_*r*_ between groups (healthy volunteers: 0.289 (0.254); PG: 0.139 (0.126); BED: 0.247 (0.232); *F*(2,34) = 1.70, *p* = 0.12) (*w*_*l*_ was not compared between groups as only healthy volunteers were tested).

We have also tested whether other computational parameters differed between groups. There were no significant differences with other parameters including learning rates, temperature or reinforcement eligibility parameter. There was a significant group difference in perseveration, or the tendency to select the same choice in the first stage irrespective of outcome (PG: 0.06 (0.14), Healthy volunteers 0.16 (0.10), BED: 0.3 (0.26) (*p* = 0.009) with posthoc analysis showing differences between PG and BED (*p* = 0.007). These findings are consistent with high perseveration scores in BED previously reported^4^.

We compared the model fits and did not show a difference between groups (negative log likelihoods (−LL): w_r: control: 142.08 (27.60); PG 154.06 (29.57); BED 137.74 (43.11); *p* = 0.46; w_l: 138.65 (37.17)). We note that the model fit for this analysis was largely similar to our existing healthy control data set (see Supplemental Materials). We also ran a supplementary analysis with [^11^C]MADAM and w_r and [^11^C]carfentanil and w_l with −LL for reward and loss included as a variable respectively with both models remaining significant (reward: *p* = ; loss: *p* = 0.007).

### PET imaging data

#### Reward

**Fig. 2:**
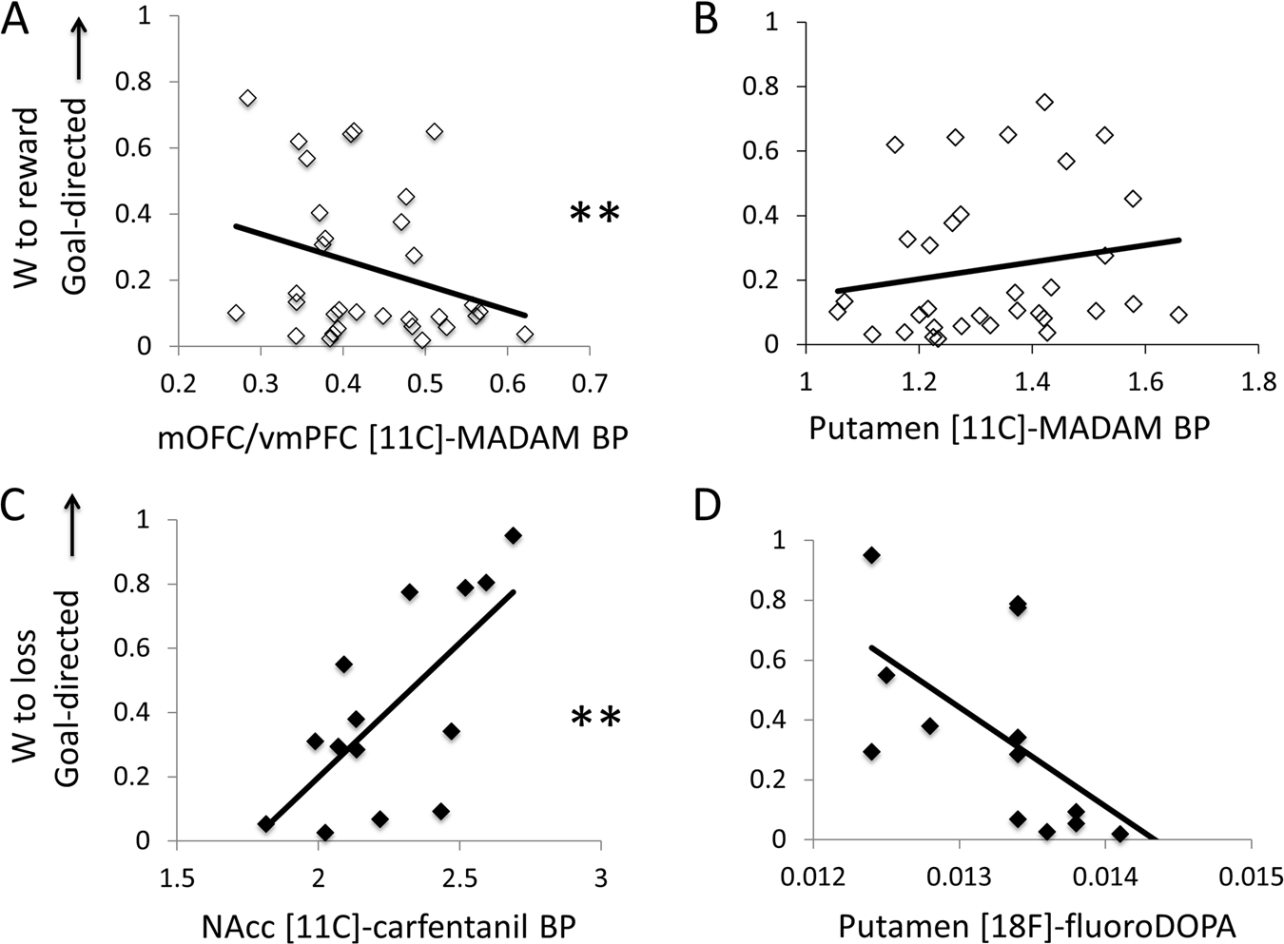
The neurochemical substrates of goal-directed control for rewards and losses. **a** Significant relationship between the relative balance of model-based and model-free control for rewards and medial orbitofrontal cortex (mOFC)/ventromedial prefrontal cortex (vmPFC) [^11^C]MADAM BP (across all participants). **b** Nonsignificant relationship between the relative balance of model-based and model-free control for rewards and putamen [^11^C]MADAM BP (across all participants). **c** Significant relationship between the relative balance of model-based and model-free control for losses and nucleus accumbens (NAcc) [^11^C]carfentanil BP (healthy controls only). **d** Nonsignificant relationship between the relative balance of model-based and model-free control for losses and putamen [^18^F]FDOPA. **p < 0.0083 (alpha Bonferroni-corrected for six multiple comparisons).

The linear regression for *w*_*r*_ (collapsed across all three groups) showed a significant relationship with [^11^C]MADAM BP (*R*^2^ = 0.330, *F* = 4.791, *p* = 0.008) (which was significant after Bonferroni correction). The NAcc was not associated with *w*_*r*_ and was subsequently removed from the model. The final model showed that *w*_*r*_ was significantly negatively correlated with vmPFC/mOFC (Beta = −0.653, *t* = −3.406, *p* = 0.002), positively correlated with putamen (Beta = 0.421, *t* = 2.352, *p* = 0.040), and marginally associated with caudate (Beta = 0.332, *t* = 1.876, *p* = 0.071) [^11^C]MADAM BP_._ In sum, greater goal-directed control (and weaker habitual control) was associated with putamen [^11^C]MADAM BP, while greater habitual control (and weaker goal-directed control) was associated with vmPFC/mOFC [^11^C]MADAM BP. There were no significant relationships between *w*_*r*_ and [^18^F]FDOPA (*R*^2^ = 0.081, *F* = 2.554, *p* = 0.121) or [^11^C]carfentanil BP (*R*^2^ = 0.008, *F* = 0.259, *p* = 0.614). See Fig. 2a, b.

#### Loss

The linear regression for *w*_*l*_ and [^11^C]carfentanil BP was significant with all regions included in the model (*R*^2^ = 0.472, *F* = 10.728, *p* = 0.007) (note *w*_*l*_ includes only healthy participants, as this version of the task was only run in healthy participants) (which remained significant after Bonferroni correction). However, the vmPFC/mOFC, caudate, and putamen were not significantly associated with *w*_*l*_, and were therefore removed from the model. In the final model, *w*_*l*_, or greater goal-directed control (or impaired habitual control) toward losses, was significantly positively correlated with bilateral NAcc [^11^C]carfentanil BP (Beta = 0.687, *t* = 2.275, *p* = 0.007). See Fig. 2c.

The linear regression for *w*_*l*_ and [^18^F]FDOPA showed only a trend (after Bonferroni correction) in the relationship between *w*_*l*_ (*R*^2^ = 0.337, *F* = 5.598, *p* = 0.037); this was no longer significant after Bonferroni correction. The vmPFC/mOFC, caudate and NAcc were not significantly associated and were removed from the model. The final model of *w*_*l*_ and bilateral putamen [^18^F]FDOPA (Beta = −0.581, *t* = −2.366, *p* = 0.037), such that higher putaminal [^18^F]FDOPA was associated with impaired goal-directed control (or greater habitual control) toward losses but critically was not significant after correction (see Fig. 2d). Given previous positive findings^13^, we also specifically tested a regression analysis with NAcc [^18^F]FDOPA for w_r and w_l, and show no significant findings (*p* = 0.98 and *p* = 0.32, respectively).

There were no significant relationships between *w*_*l*_ and [^11^C]MADAM BP (*R*^2^ = 0.038, *F* = 0.475, *p* = 0.504).

#### Valence specificity and behavioural measures of model-based and model-free control

To assess specificity of the effect of the tracer on valence we reran the multiple regression analysis controlling for the opposing valence. As there was no evidence of multicollinearity between w for gain and loss (Tolerance = 0.74, VIF = 1.34), we conducted a secondary analysis of the regression analysis for [^11^C]MADAM and w_r including w_l into the model. The overall model including caudate and vmPFC/mOFC (but not putamen) remained significant at *p* = 0.014 (caudate *p* = 0.018; vmPFC/mOFC *p* = 0.006). Similarly, the regression analysis for [^11^C]carfentanil and w_l including w_r into the model also remained significant at p = 0.007 with only NAcc in the model (NAcc *p* = 0.007). The regression analysis for [^18^F]FDOPA and w_l including w_r into the model also showed an overall model *p* value of 0.04 with only putamen in the model (putamen *p* = 0.04). Put together, these secondary findings highlight the specificity of [^11^C]MADAM and vmPFC/mOFC for w_r and [^11^C]carfentanil and NAcc for w_l.

For the purposes of exploring the trade-off between goal-directed and habitual effects and the relationship with neurotransmitter levels, we conducted supplementary analyses with the behavioural based model-based and model-free control as the independent variables rather than w. For [^11^C]MADAM and reward, there was no significant relationship with either model-based on model-free control. For [^11^C]carfentanil and loss, greater model-based control was significantly associated with a model (*p* = 0.019) with a positive correlation with NAcc [^11^C]carfentanil BP (*t* = 3.24, *p* = 0.008); and greater model-free control was significantly associated with a model (*p* = 0.01) with a negative correlation with NAcc [^11^C]carfentanil BP (*t* = −3.04, *p* = 0.01).

## Discussion

We reveal a differential role for prefrontal and striatal serotoninergic systems in mediating the balance of goal-directed and habitual control in the reward domain: lower mOFC/vmPFC, but higher putamen [^11^C]MADAM BP correlated with a shift toward goal-directed control; however, the latter relationship was not specific when controlled for the opposing valence (loss). In the loss domain, we also find a differential relationship between opioidergic systems and both a positive correlation with goal-directed control and a negative correlation with NAcc [^11^C]carfentanil BP.

### Opioid peptides in goal-directed control

In the loss domain, we also found a positive relationship between the opioidergic system and goal-directed control and a negative relationship with habit-directed control. Here, greater NAcc [^11^C]carfentanil BP may reflect either greater MOR density or lower endogenous synaptic peptide opioid levels, which compete for binding with [^11^C]carfentanil. These findings are consistent with preclinical evidence suggesting blockade of endogenous opioid activity in rodents by the competitive opioid receptor antagonist naloxone during acquisition learning of food rewards shifts behavior toward habitual control, and decreases sensitivity to changes in the value of reward^14^. This effect was restricted to the acquisition of goal-directed actions, and not during performance in the test phase, suggesting a specific effect of MOR antagonism during goal-directed learning. An alternate explanation lies in the effect of opioids on aversive processing: opioids decrease pain ratings particularly in the expectation of pain relief^35^, and decrease non-painful aversive responses such as conditioned aversion in rodents^36^. In healthy humans, blocking MOR with naloxone during a gamble task increased the subjective aversive ratings to monetary loss outcomes^36^. Furthermore, naloxone increases blood oxygen level-dependent activity during loss outcomes in caudal and subgenual cingulate, bilateral insula, thalamus, and visual cortex; caudal cingulate activity correlates with aversive ratings^36^. Thus, in our data, an alternate plausible explanation may be that endogenously lower opioid peptides enhances the aversiveness of monetary loss, thus improving goal-directed control to losses. Note that although MOR stimulation is associated with striatal dopamine release via GABAergic mechanisms in the ventral tegmental area^37^, we did not observe any relationship between [^18^F]FDOPA and goal-directed control in our study, nor any relationship between [^18^F]FDOPA and [^11^C]MADAM or [^11^C]carfentanil BP.

### A differential role for prefrontal and striatal serotonergic systems

Perhaps the most interesting finding emerging from our study is a potential differential relationship between prefrontal and striatal serotonergic systems in mediating the balance between goal-directed and habitual control. In rodents, decreasing forebrain 5-HT and systemic 5HT2C antagonism enhances compulsive cocaine seeking, an effect which was reversed by both a 5HT2C agonist and a selective serotonin reuptake inhibitor^14^. Furthermore, overexpression of dorsolateral striatal 5-HT6 receptors decreases habitual control in rodents^15^. In healthy humans, central serotonin depletion enhances habitual responding^17^ and impairs goal-directed control to rewards, while enhancing goal-directed control to losses^6^. Patients with obsessive–compulsive disorder (with putative impairments in serotonergic function) show impaired goal-directed control for rewards and enhanced goal-directed control for losses^33^.

It is worth noting that SERT BP is interpreted in terms of serotonin terminal density (SERT density), which can be either primary or adaptive in response to endogenous serotonin level changes; these have opposing implications for serotonin levels. If we presume that low SERT BP reflects fewer serotonergic terminals, and hence lower serotonergic activity, our prefrontal results support previous findings that low forebrain serotonin in rodents enhances compulsive cocaine seeking^14^ and central serotonin depletion in healthy humans impairs goal-directed control and shifts behavior toward habitual responding for rewards^17^. However, we fail to confirm previous studies showing valence-dependent effects on serotonin on goal-directed processing^6^ (we show no effect in the loss domain), which is inconsistent with previous work showing a key role of serotonin in loss or punishment processes^6,38^.

### Presynaptic dopamine synthesis and habitual control

There are conflicting preclinical and human reports regarding dopaminergic function in goal-directed and habitual control. In rodents, pharmacologically enhancing dopamine (with amphetamine) accelerates habit formation^7^, a process reversed by D1 antagonism (but enhanced by D2 antagonism)^39^; selective nigrostriatal dopaminergic lesions impair habit formation^8^. In contrast, in healthy humans, depletion of the dopamine precursor *increases* habitual control^9^. The severity of Parkinson’s disease, characterized by dopaminergic deficits, is associated with impairments in goal-directed control^9^; patients tested off-medication show impaired goal-directed control. Pharmacological enhancement of dopamine with levodopa increases goal-directed control in both Parkinson’s disease patients^11^ and healthy controls^10^; although note this may not generalize to all individuals, as a more recent study found that levodopa decreased habitual control, with increases in goal-directed control only seen in individuals with a high working memory capacity^12^. Nevertheless, greater ventral striatal presynaptic dopamine synthesis, measured using F-DOPA PET, correlates with greater goal-directed control^13^. These human studies contrast with the preclinical literature^9,11^ and may be related to task differences such as overtraining in rodent relative to human studies, lack of anatomical specificity of dopaminergic medication challenges in humans, or overlap of neural substrates underlying goal-directed and habitual control^3^.

Our observations in healthy controls are more consistent with the preclinical literature: we show a marginal relationship between greater presynaptic dopamine synthesis in putaminal regions and habitual control in the loss domain which was no longer significant after multiple correction. A previous study showed a weak positive relationship between [^18^F]FDOPA and goal-directed control to rewards in 29 healthy controls^13^. However, we were unable to replicate these findings. Our lack of positive findings in the reward domain should be interpreted with caution, as we may not have had adequate power to replicate this effect. However, the negative relationship we observed with in the loss domain could imply a differential relationship between the role of dopamine in goal-directed and habit control for rewards versus losses.

### Limitations

Our study is the first to investigate the role of three neurochemical systems—serotonergic, dopaminergic, and opioidergic—in goal-directed and habitual control. As such, while we reveal a number of interesting potential relationships, we are limited by both inherent ambiguities in the interpretation of BP effects, and a relative dearth of similar investigations in humans. Furthermore, while our study was adequately powered for within-group comparisons, our lack of a group effect may simply reflect inadequate power to detect between-group differences. This lack of power could also account for our lack of group differences on our behavioural measure (*w*_*r*_); previous studies have shown this measure to be generally compromised across disorders of compulsitivity^3–5^.

We also tested whether other computational parameters differed between groups. There were no significant differences with other parameters including learning rates, temperature or reinforcement eligibility parameter. There was a significant group difference in perseveration, the tendency to select the same choice in the first stage irrespective of outcome (PG: 0.06 (0.14), healthy volunteers: 0.16 (0.10), BED: 0.3 (0.26); *p* = 0.009) with a posthoc analysis showing differences between PG and BED (*p* = 0.007). Despite our small sample size of patients with BED, we replicate the finding of increased perseveration irrespective of outcome, which we previously reported in a much larger sample: patients with BED showed increased perseveration on this task compared to obese participants without BED^4^. This fits in with a larger experimental and clinical literature reporting cognitive inflexibility in BED: patients with BED show decreased cognitive flexibility on a neuropsychological battery compared to either healthy controls or patients with anorexia^40^ (for a review of the literature, see ref. ^41^). This impairment in cognitive flexibility could contribute to the symptoms of BED by making patients less able to change their decisions about food consumption after changing environmental outcomes (e.g., the food losing value after satiety, or nausea or discomfort as a result of overeating).

In addition, our findings in the reward domain were strengthened by the inclusion of both healthy controls and a transdiagnostic psychiatric population; in contrast, our findings in the loss domain were limited to the healthy population. In future, it would be essential to extend our transdiagnostic results to the loss domain, but also investigate samples large enough to characterize any between-group relationships in each neurochemical system and its role in goal-directed and habitual control.

## Conclusions

We highlight a potential role for dopaminergic, opioidergic and serotonergic mechanisms in arbitrating between behavioral controllers. In the reward domain, we showed a differential role for prefrontal and striatal serotonergic mechanisms, which were associated with habitual and goal-directed control, respectively. In the loss domain, we found the NAcc opioidergic system was positively associated with goal-directed control, and more tentatively, that the putaminergic dopaminergic system was associated with habitual control. These findings begin to reveal the complex neurochemical substrates of a key aspect of decision-making. Uncovering these mechanisms could be crucial to developing interventions that target these behavioural strategies in the context of psychiatric disorders.

## Supplementary information

Supplemental Material
