# Supplementary material for: The neurochemical substrates of habitual and goal-directed control
Source: Transl Psychiatry. 2020 Mar 3;10:84. doi: 10.1038/s41398-020-0762-5 (PMC7054261; doi:10.1038/s41398-020-0762-5)
Supplement: Supplementary file 1 — Supplemental Material [file 41398_2020_762_MOESM1_ESM.docx]

Supplemental materials

**The neurochemical substrates of habitual and goal-directed control**

*Supplemental analysis of existing healthy control data on shortened task*

To support our decision to use a shorter version of the sequential learning task, we analysed 172 healthy controls (from our existing database) to show the following: 1 block (67 trials): w=0.29 (0.22), negative log likelihood (-LL)=67.88 (17.66); 2 blocks (134 trials): w=0.33 (0.24), -LL=138.38 (30.21); 3 blocks (201 trials): w=0.34 (0.24), -LL=208.64 (40.06). The Pearson correlation between 2 blocks and 3 blocks for w was Pearson correlation coefficient=0.78 (p<0.0001) and for -LL was Pearson correlation coefficient=0.96 (p<0.0001) indicating a strong correlation. The model fit (-LL) appeared better for 3 relative to 2 blocks but the scores of w were relatively equivalent for 2 and 3 blocks with high within individual correlations between both blocks 2 and 3 for w and model fits.
